# The effects of long-acting bronchodilators on total mortality in patients with stable chronic obstructive pulmonary disease

**DOI:** 10.1186/1465-9921-11-56

**Published:** 2010-05-11

**Authors:** Agnes Kliber, Larry D Lynd, Don D Sin

**Affiliations:** 1 Department of Medicine (Respiratory Division), University of British Columbia, 6040 Iona Drive, Vancouver, V6T 2E8, Canada; 2 Faculty of Pharmaceutical Sciences, University of British Columbia, 6040 Iona Drive, Vancouver, V6T 2E8, Canada; 3 Providence Heart and Lung Institute, St. Paul's Hospital, 1081 Burrard Street, Vancouver, Canada, V6Z 1Y6; 4 James Hogg Research Laboratories, 1081 Burrard Street, Vancouver, British Columbia, Canada, V6Z 1Y6

## Abstract

**Background:**

Chronic obstructive pulmonary disease (COPD) is the 4^th ^leading cause of mortality worldwide. Long-acting bronchodilators are considered first line therapies for patients with COPD but their effects on mortality are not well known. We performed a comprehensive systematic review and meta-analysis to evaluate the effects of long-acting bronchodilators on total mortality in stable COPD.

**Methods:**

Using MEDLINE, EMBASE and Cochrane Systematic Review databases, we identified high quality randomized controlled trials of tiotropium, formoterol, salmeterol, formoterol/budesonide or salmeterol/fluticasone in COPD that had a follow-up of 6 months or longer and reported on total mortality. Two reviewers independently abstracted data from the original trials and disagreements were resolved by iteration and consensus.

**Results:**

Twenty-seven trials that included 30,495 patients were included in the review. Relative risk (RR) for total mortality was calculated for each of the study and pooled together using a random-effects model. The combination of inhaled corticosteroid (ICS) and long-acting beta-2 agonist (LABA) therapy was associated with reduced total mortality compared with placebo (RR, 0.80; p = 0.005). Neither tiotropium (RR, 1.08; p = 0.61) nor LABA by itself (RR, 0.90; p = 0.21) was associated with mortality.

**Conclusions:**

A combination of ICS and LABA reduced mortality by approximately 20%. Neither tiotropium nor LABA by itself modifies all-cause mortality in COPD.

## Introduction

Chronic obstructive pulmonary disease (COPD) affects more than 300 million people worldwide [1]. It is currently the 4^th ^leading cause of mortality accounting for nearly 3 million deaths annually and is the only major cause of mortality that is increasing in both the developed and developing countries [2]. By 2020, it will become the 3^rd ^leading cause of death (accounting for 5 million deaths per year) and the 5^th ^leading causing of disability worldwide [2]. Expert guidelines recommend the use of long-acting bronchodilators as first-line therapies for patients with persistent symptoms [3,4]. However, their effect on mortality remains controversial. A previous meta-analysis suggested that inhaled long-acting anticholinergic bronchodilators had no effect on total mortality [5]. On the other hand, a secondary analysis of the UPLIFT trial suggested a mortality benefit [6]. Similarly, although the TORCH trial suggested a modest mortality benefit with inhaled corticosteroid/long-acting beta-2 agonist combination (ICS/LABA), meta-analyses suggested that they may only reduce mortality when compared to placebo [7] or ICS alone [8] but not to LABA alone [7]. However, there were several limitations to the prior meta-analyses, which may have led to some of the discordant findings. First, the prior meta-analysis on tiotropium did not include data from the recently completed UPLIFT trial. Second, prior meta-analyses did not address the effect of LABA on total mortality, making it difficult to assess whether or not LABA can be used as a reasonable comparator for ICS/LABA. Third, the findings from the ICS/LABA on mortality are dominated by data from one trial (i.e. TORCH), raising doubts about the robustness of the results from previous meta-analyses. Fourth, and most importantly, many of the previous trials of ICS/LABA used a factorial design. However, none of these studies had sufficient power to assess interactions between treatments or to adjust for multiple comparisons. From a methodological perspective, it is essential that the active treatment drugs be compared against one (primary) reference group (and not to each other) unless adjustments are made for multiple comparisons [9]. To address these limitations and to determine the effects of these drugs on total mortality in COPD, we performed a systematic review and meta-analysis with and without TORCH for ICS/LABA and inclusive of UPLIFT for tiotropium. Importantly, to maintain statistical integrity, for trials that used a factorial design, we determined survival effects of the primary active treatment drug against the principal comparator group identified *a priori *in each of the individual studies.

## Methods

### Data Sources and Searches

We examined the relationship of tiotropium, a long-acting anticholinergic, as well as formoterol and salmeterol, which are long-acting beta-2 agonists, by themselves or in combination with an inhaled corticosteroid to all-cause mortality. Using MEDLINE, EMBASE and Cochrane Systematic Review databases, we conducted a detailed literature search to identify high-quality randomized controlled trials of tiotropium, formoterol, salmeterol, formoterol/budesonide or salmeterol/fluticasone in patients with stable COPD in which total mortality was reported. We supplemented the electronic search by reviewing the bibliographies of selected articles, examining review articles on this topic and contacting experts in the field. Studies in abstract form were included only if the methods and results could be adequately analyzed.

### Study Selection

We restricted the search to studies that were conducted in adults (>19 years of age), had follow-up of 6 months or greater, and were published in the English language with a Jadad score of 3 or greater [10]. We restricted the duration to 6 months to ensure that patients had a reasonable window of exposure to the drugs. We excluded trials in which there were no deaths. The details of the search are provided in Additional File 1.

### Data Extraction and Quality Assessment

Data were abstracted from each trial by 2 authors (A.K, D.D.S) independently using a standardized data abstraction form. Any discrepancies were resolved by iteration and consensus. The primary endpoint was total mortality (regardless of the cause). Disease specific mortality was not determined as assigning causality to deaths in COPD is problematic and fraught with errors [11]. The trials were stratified according to the study drug and to the main comparator group. For analytic purposes, the active treatment compound (i.e. tiotropium, formoterol, salmeterol or formoterol/budesonide or salmeterol/fluticasone) was compared against the main reference group. In most cases, the main reference group was placebo; however, we also included studies in which the main comparator was another active drug (e.g. tiotropium or salmeterol). Quality of the trials was assessed using the QUOROM guidelines as well as using the Jadad scale [10].

### Data Synthesis and Analysis

The results were analyzed by intention-to-treat whenever possible. To maintain the statistical integrity of the original trial, for studies that used a factorial design, we determined the mortality rate of the active treatment drugs against one (primary) reference group (e.g. placebo) that were identified *a priori*. This mitigated the possibility of *post hoc *analyses. To be conservative, a DerSimonian and Laird random-effects model was used to pool the results of individual trials together. The results are reported as relative risks (RR) and 95% confidence intervals (CI). Heterogeneity of results across individual studies was examined using a chi-square test. All analyses were conducted using RevMan version 5.0 (the Cochrane Collaboration, Oxford, England).

## Results

**Figure 1:**
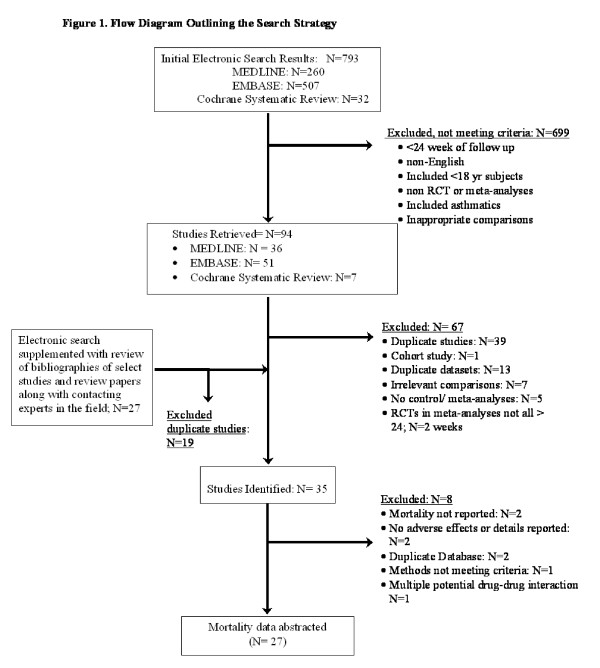
**Flow Diagram Outlining the Search Strategy**.

**Figure 2:**
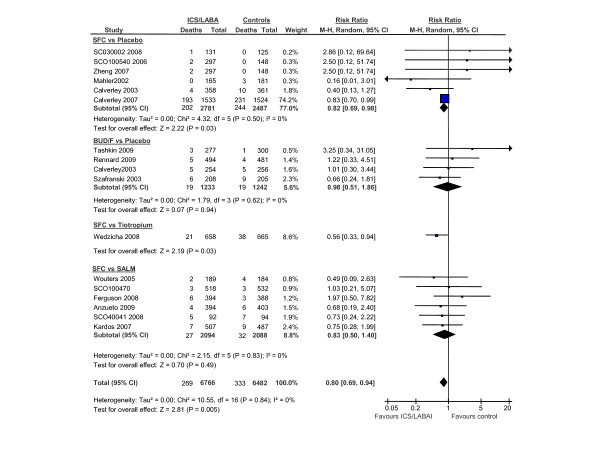
**The Effects of Inhaled Corticosteroid/Long-Acting Beta-2 Agonist Combination on Total Mortality**. Abbreviations: BUD/F, budesonide/formoterol combination; CI, confidence interval; ICS/LABA, inhaled corticosteroids/long-acting beta-2 agonist combination; M-H, Mantel-Hanzel; SALM, salmeterol; SFC, salmeterol/fluticasone combination

**Figure 3:**
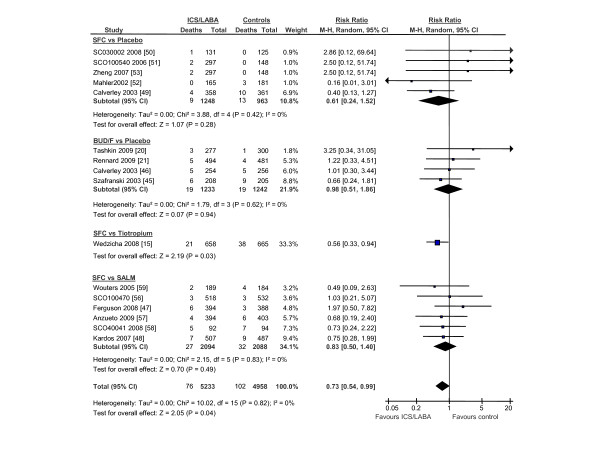
**The Effects of Inhaled Corticosteroid/Long-Acting Beta-2 Agonist Combination on Total Mortality Excluding Calverley et al's Trial **[13]. Abbreviations: BUD/F, budesonide/formoterol combination; CI, confidence interval; ICS/LABA, inhaled corticosteroids/long-acting beta-2 agonist combination; M-H, Mantel-Hanzel; SALM, salmeterol; SFC, salmeterol/fluticasone combination

**Figure 4:**
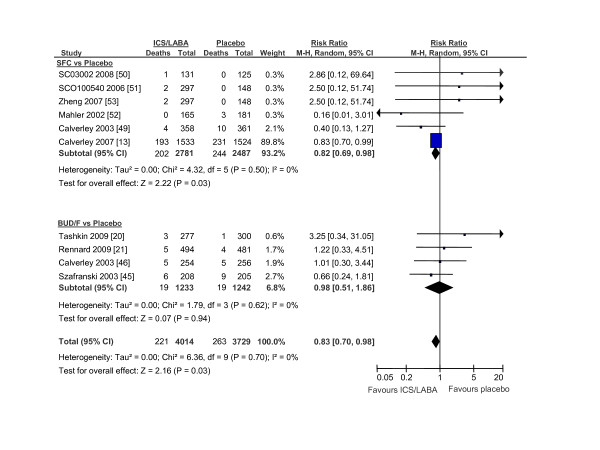
**The Effects of Inhaled Corticosteroid/Long-Acting Beta-2 Agonist Combination on Total Mortality Using Clinical Trials That Used Placebo-Treated Patients As The Main Comparator**. Abbreviations: BUD/F, budesonide/formoterol combination; CI, confidence interval; ICS/LABA, inhaled corticosteroids/long-acting beta-2 agonist combination; M-H, Mantel-Hanzel; SALM, salmeterol; SFC, salmeterol/fluticasone combination

The search results are shown in figure 1. The baseline patient characteristics of the selected studies are summarized in Table 1. We identified 6 trials that compared salmeterol/fluticasone combination against placebo (n = 2781 in active treatment vs 2487 in placebo), 4 trials that compared formoterol/budesonide against placebo (n = 1233 vs n = 1242), 1 trial that compared salmeterol/fluticasone against tiotropium (n = 658 vs 665) and 6 trials that compared salmeterol/fluticasone against salmeterol by itself (n = 2094 vs n = 2088). One trial was excluded as treatment with salmeterol/fluticasone or salmeterol alone was in addition to tiotropium, which could have led to significant drug to drug interactions [12]. The collective results of inhaled corticosteroid/long-acting beta-2 agonist combination are summarized in figure 2. In total, there were 269 deaths in the inhaled corticosteroid (ICS)/long acting beta-2 agonist (LABA) arm (n = 6766) and 333 deaths in the reference group (n = 6482) for a relative risk of 0.80 (95% CI, 0.69 to 0.94; p = 0.005) in favor of the active treatment group. The results were largely driven by data from Calverley *et al*, which accounted for 74% of the total weight [13]. The data, however, were robust to the exclusion of Calverley *et al*'s study. Its exclusion resulted in a similar risk estimate in favor of ICS/LABA combination (RR, 0.73; 95% CI, 0.54 to 0.99; p = 0.04) (figure 3). The comparison between ICS/LABA and placebo (excluding studies that did not use a placebo comparator) was also significant (RR, 0.83; 95% CI, 0.70 to 0.98; p = 0.03; see figure 4).

**Table 1:** Summary Of Clinical Trials That Were Included In This Analysis

Author	N	Drug 1	Drug 2	Comparator	Follow-up	Mean Age (SD)	% Men	Mean FEV_1 _(SD)	% Current Smokers	Jadad Score	Prohibited COPD Drugs
Casaburi 2002[39]	921	TI (18 ug OD)	None	PL	49 weeks	64 (9)	76	1.02 (0.44)	NA	3	LABA/other anticholinergics

Chan 2007[40]	913	TI (18 ug OD)	None	PL	48 weeks	66 (9)	60	0.97 (0.38)	32	3	other anticholinergics, oral beta agonists

Niewoehner 2005[41]	1829	TI (18 ug OD)	None	PL	6 months	68 (10)	99	1.04 (0.4)	30	3	other anticholinergics; oral CS >20 mg/d

Tashkin 2008[35]	5993	TI (18 ug OD)	None	PL	9 months	65 (8)	75	1.10 (0.4)	29	4	other anticholinergics

Tonnel 2008[42]	554	TI (18 ug OD)	None	PL	6 months	64 (10)	86	1.36 (0.46)	75	4	other anticholinergics

Vincken 2002[14]	535	TI (18 ug OD)	None	IP (40 ug QID)	6 months	64 (8)	85	1.22 (0.43)	NA	3	LABAs, other anticholinergics

Brusasco 2003[43]	1207	TI (18 ug OD)	SALM (50 ug BID)	PL	6 months	64 (9)	76	1.10 (0.39)	NA	3	Insufficient details provided

Vogelmeier 2008[44]	640	TI (18 ug OD)	FORM (10 ug BID)	PL	24 weeks	63 (9)	78	1.52 (0.39)	NA	3	Insufficient details provided

Tashkin 2008[20]	1148	BUD/FORM (320 ug/9 ug BID)	FORM (9 ug BID)	PL	6 months	63 (10)	67	1.04 (0.41)	41	4	All prohibited except salbutamol

Szafranski 2003[45]	614	BUD/FORM (320 ug/9 ug BID)	FORM (9 ug BID)	PL	12 months	64 (NA)	80	0.98 (NA)	35	3	Only study drugs allowed

Calverley 2003[46]	765	BUD/FORM (320 ug/9 ug BID)	FORM (9 ug BID)	PL	12 months	64 (NA)	76	0.99 (0.33)	34	3	ICS, all bronchodilators except terbutaline, leukotriene modifiers,

Rennard 2009[21]	1470	BUD/FORM (320 ug/9 ug BID)	FORM (9 ug BID)	PL	12 months	63 (9)	64	1.01 (0.43)	42	3	All drugs except salbutamol

Ferguson 2008[47]	782	SALM/FLU (50 ug/250 ug BID)	None	SALM (50 ug BID)	12 months	65 (9)	56	0.94 (0.36)	40	3	ICS, LABA, TI

Kardos 2007[48]	998	SALM/FLU (50 ug/500 ug BID)	None	SALM (50 ug BID)	44 weeks	63 (8)	76	1.13 (0.41)	42	3	regular oral CS, LABA and TI

Wedzicha 2008[15]	1323	SALM/FLU (50 ug/500 ug BID)	None	TI 18 ug OD	24 months	64 (NA)	83	1.12 (NA)	38	4	All drugs except salbutamol

Calverley 2003[49]	1091	SALM/FLU (50 ug/500 ug BID)	SALM (50 ug BID)	PL	12 months	64 (NA)	73	1.26 (0.48)	52	5	Oral or inhaled CS or LABAs

SCO30002 2008 [50]	387	SALM/FLU (25 ug/250 ug BID)	FP (250 ug BID)	PL	12 months	65 (9)	82	1.54 (NA)	NA	3	Not specified

SCO100540 2006[51]	445	SALM/FLU (50 ug/500 ug BID)	None	PL	6 months	66 (8)	89	1.05 (0.37)	NA	3	Not specified

Mahler 2002[52]	506	SALM/FLU (50 ug/500 ug BID)	SALM (50 ug BID)	PL	24 weeks	62 (NA)	64	1.27 (NA)	50	3	Bronchodilators except salbutamol, oral CS

Calverley 2007[13]	4633	SALM/FLU (50 ug/500 ug BID)	SALM (50 ug BID)	PL	36 months	65 (8)	76	1.12 (0.4)	43	4	CS, LABA

Zheng 2007[53]	445	SALM/FLU (50 ug/500 ug BID)	NA	PL	24 weeks	66 (8)	89	1.05 (NA)	22	4	ICS, LABA

Stockley 2006[54]	634	SALM (50 ug BID)	NA	PL	12 months	62 (9)	77	1.30 (0.5)	40	4	TI or LABA

SCO30002 2008[55]	256	SALM/FLU (50 ug/500 ug BID)	FLU (500 ug BID)	PL	52 weeks	64 (NA)	82	1.50 (NA)	NA	3	Not specified

SCO100470 2006 [56]	1050	SALM/FLU (50 ug/250 ug BID)	None	SALM (50 ug BID)	6 months	64 (9)	78	1.67 (0.46)	NA	3	Not specified

Anzueto 2009[57]	797	SALM/FLU (50 ug/250 ug BID)	None	SALM (50 ug BID)	12 months	65 (NA)	54	1.17 (0.51)	42	4	All prohibited except salbutamol and ipratropium

SCO40041 2008 [58]	186	SALM/FLU (50 ug/250 ug BID)	None	SALM (50 ug BID)	3 years	66 (9)	61	NA	NA	3	Not specified

Wouters 2005 [59]	373	SALM/FLU (50 ug/500 ug BID)	None	SALM (50 ug BID)	12 months	64 (8)	74	1.41 (0.48)	37	4	All prohibited except salbutamol, ipratropium and xanthines

Abbreviations:

BID, twice daily; CS, corticosteroids; FEV_1_, forced expiratory volume in one second; FLU, fluticasone; FORM, formoterol; ICS, inhaled corticosteroids; IP, ipratropium bromide; LABA, long acting bronchodilators, N = total sample size; NA, not available; OD, once daily; PL, placebo; SALM, salmeterol; SD, standard deviation; TI, tiotropium

**Figure 5:**
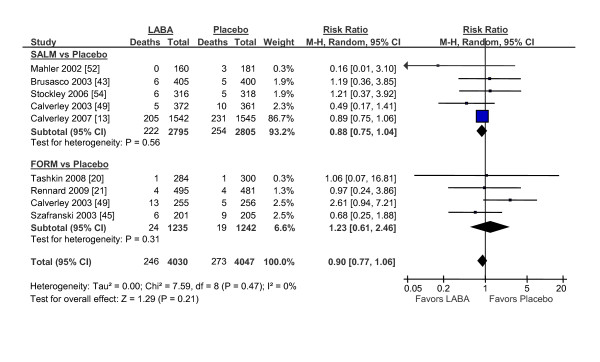
**The Effects of Long-Acting Beta-2 Agonists on Total Mortality**. Abbreviations: CI, confidence interval; FORM, formoterol; LABA; long-acting beta-2 agonists; M-H, Mantel-Hanzel; SALM, salmeterol

We identified 5 trials that compared salmeterol against placebo. There were 222 deaths in the salmeterol group (n = 2795) and 254 deaths in the placebo group (n = 2805) for a relative risk of 0.88 (95% CI, 0.75 to 1.04; p = 0.13) (figure 5). There were 4 trials that compared formoterol against placebo. In these studies, there were 24 deaths in the formoterol group (n = 1235) and 19 deaths in the placebo group (n = 1242) for a relative risk of 1.23 (95% CI, 0.61 to 2.46; p = 0.57). In total, the long-acting beta-2 agonists by themselves did not significantly alter total mortality in COPD (RR, 0.90; 95% CI, 0.77 to 1.06; p = 0.21).

**Figure 6:**
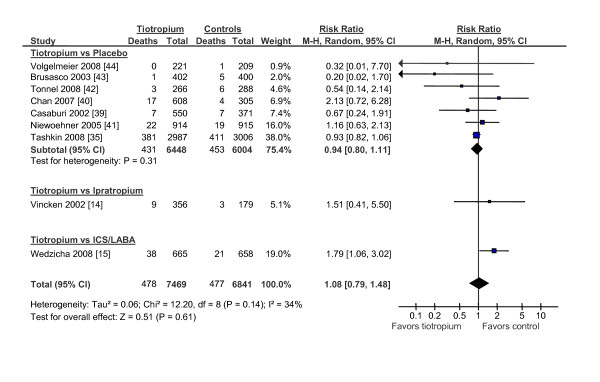
**The Effects of Tiotropium on Total Mortality**. Abbreviations: CI, confidence interval; ICS/LABA, inhaled corticosteroids/long-acting beta-2 agonist combination; M-H, Mantel-Hanzel

**Figure 7:**
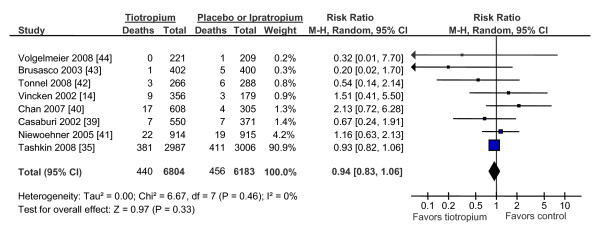
**The Effects of Tiotropium on Total Mortality Using Clinical Trials That Used Placebo or Ipratropium Bromide As The Main Comparator**. Abbreviations: CI, confidence interval; ICS/LABA, inhaled corticosteroids/long-acting beta-2 agonist combination; M-H, Mantel-Hanzel

We identified 9 clinical trials that compared tiotropium against placebo but one study reported no deaths and data from one of the studies overlapped substantially with another, leaving 7 clinical trials for analysis (figure 6). In all, there were 431 deaths in the tiotropium group and 453 deaths in the placebo group for a relative risk of 0.94 (95% CI, 0.80 to 1.11; p = 0.46). There was one study that compared tiotropium against ipratropium (RR, 1.51; 95% CI, 0.41 to 5.50; p = 0.53) [14] and one that compared tiotropium against salmeterol/fluticasone combination (RR, 1.79; 95% CI, 1.06 to 3.02; p = 0.03) [15]. In sum, tiotropium was not associated with total mortality (RR, 1.08; 95% CI, 0.79 to 1.48; p = 0.61). As a sensitivity analysis, we excluded studies that compared tiotropium against a comparator other than placebo or ipratropium bromide and repeated the analysis. This made no material impact on the results (figure 7). Tiotropium was not associated with total mortality (RR, 0.94; 95% CI, 0.83 to 1.06; p = 0.33).

## Discussion

The most important findings from the present meta-analysis were that 1) inhaled corticosteroids (ICS) in combination with a long-acting bronchodilator (LABA) were associated with a ~20% reduction in total mortality; whereas LABAs or long-acting anticholinergics by themselves did not alter mortality; and 2) these data were robust to the inclusion or exclusion of the TORCH and UPLIFT trials.

These findings are largely in keeping with previous observational studies, which have shown for the most part enhanced survival with the use of ICS/LABA combinations and a lack of survival benefits of short or long acting bronchodilators by themselves [16-18]. However, our findings appear discordant with a recent meta-analysis published by Rodrigo and colleagues [19], which failed to find a significant difference in total mortality between those treated with ICS/LABA and those treated with LABA only (though the point estimate was 0.90 in favor of ICS/LABA). However, this study excluded trials that did not have a LABA arm and failed to capture more recently published clinical trials (e.g. studies by Tashkin et al[20] and Rennard et al[21]). By adding these additional studies, the present meta-analysis had greater statistical power to determine the relationship of ICS/LABA combination to total mortality. More importantly, in the present meta-analysis, we compared the active treatment groups (i.e. ICS/LABA or LABA or tiotropium) against the primary reference group of the trial in order to preserve the integrity of the original trial design and avoid the problem of multiple comparisons and *post hoc *analyses. Thus, for the trials that used a 2 × 2 factorial design, we compared the mortality effects of the active treatment arm against the main reference group of the trial (which in most cases was placebo) as the original trials did not have sufficient power to assess interactions between the groups or to correct for multiple comparisons [22].

The mechanism by which ICS/LABA combination reduces total mortality in COPD is uncertain. It is now well recognized that COPD is an inflammatory disorder, characterized by persistent lung and systemic inflammation, which intensifies with disease progression and during clinical exacerbations [23,24]. Once COPD develops, the inflammatory response continues to persist many years after smoking cessation [25]. Although the inflammatory process in COPD may be relatively insensitive to the actions of glucocorticoids, the addition of a long-acting beta-2 agonist to an inhaled corticosteroid appears to amplify their anti-inflammatory effects both *in vitro *[26] and *in vivo *[27,28]. For instance, Bourbeau and colleagues found that 3 months of therapy with salmeterol/fluticasone combination attenuated lung inflammation, as characterized by a reduction in the number of CD8 positive and CD68 positive cells in the airways of patients with severe, stable COPD; whereas fluticasone by itself had no effect [28]. Similarly, Barnes and colleagues observed a significant reduction in the expression of inflammatory biomarkers in the bronchial biopsies and sputum of COPD patients treated with salmeterol/fluticasone combination compared to those treated with placebo [27]. These data have been replicated and extended by Lapperre and colleagues, who showed that salmeterol/fluticasone therapy for 30 months reduced lung inflammation, attenuated the rate of decline in lung function, and improved bronchial responsiveness compared to salmeterol alone or placebo[29]. Inhaled corticosteroid/long acting beta-2 agonist combination may also attenuate the systemic inflammatory response in COPD [30], which is associated with morbidity and mortality [31,32].

In addition to their anti-inflammatory effects, combination therapy results in greater bronchodilation than that achieved by the individual mono-components [13]. However, the combined bronchodilatory effects of inhaled corticosteroid/beta-2 agonists is no better than that achieved with tiotropium alone in COPD [15]. Despite this, the combination therapy results in superior health status, and reduced mortality compared with tiotropium alone [15], suggesting that mechanisms other than bronchodilation and lung deflation are involved in the mortality benefits of combination therapy.

There were limitations to the present study. We did not have access to individualized data; thus, we could not adjust for potential confounders. However, to mitigate confounding, we chose large randomized controlled trials, which were of high quality (Jadad Score of 3 or greater) and had a reasonable duration of follow-up (6 months or greater), detailed accounting of all randomized patients in the study and reported excellence balance in terms of patient characteristics and clinical status between the active treatment and comparator arms. Secondly, there was some heterogeneity in the doses and drugs that were evaluated across the trials. We addressed this issue by grouping the studies together, stratified according to the drug formulation and dose and used a conservative method of pooling the data (i.e. a random effects model). Thirdly, we assessed total but not disease specific mortality. We did not evaluate disease specific mortality because assigning causality to deaths in COPD is problematic [11]. Moreover, certain drugs have been associated with increased risk of non-COPD related health events such as pneumonia [33] and stroke [34], which could have been missed by focusing on COPD-specific mortality alone. Fourthly, we did not evaluate the effects of inhaled corticosteroids on total mortality because recent studies have established that these drugs do not impact on overall mortality and expert guidelines in general do not recommend inhaled corticosteroids as standalone therapies for COPD [13,33]. Fifthly, some recent trials were performed on the background of bronchodilators, inhaled corticosteroids or both, which may have diluted the possible mortality benefits of the drug in question. This may be of particular concern in the most recent tiotropium trial in which a majority of study patients were taking ICS, LABA or both at the time of recruitment [35]. Additionally, none of the studies included in this meta-analysis except for Calverley *et al*.' s study [13] was powered on mortality. As such, patients with complex or life-threatening co-morbidities were generally excluded from these trials, which likely reduced the statistical power of the present study and limited the generalizability of the findings to patients with multiple co-morbidities. Another important consideration was the differential drop-out rate between the active treatment and the comparator arms of the study. Collectively, the patients in the comparator arm were more likely to drop-out of the trials compared with those who were assigned to active treatment arm (38% versus 30%; p < .0001). Although the precise effects of differential drop-out rate are not fully known, it may have biased the results in favor of the comparator arm, as patients who drop out are generally sicker, less motivated and have poorer prognosis than those who remain in the study [36].

COPD is a worldwide epidemic affecting ~10% of adults 40 years of age and older and accounting for more than 3 million deaths annually. In China alone, there will be nearly 1.5 million deaths this year from COPD [37]. Discouragingly, over the next 20 years, the worldwide mortality from COPD will double [38]. The totality of data from many large, randomized clinical trials indicates that the combination of inhaled corticosteroids and long-acting beta-2 agonists prolongs survival in COPD but long-acting beta-2 agonists and tiotropium by themselves do not. The survival effect, however, is fairly modest and suggests a pressing need for additional pharmacotherapies that can reduce the overall mortality in COPD, which in less than 10 years will be the 3^rd ^leading cause of death worldwide.

## Abbreviations

BUD/F: budesonide/formoterol combination; CI: confidence interval; COPD: chronic obstructive pulmonary disease; ICS: inhaled corticosteroid; LABA: long-acting beta-2 agonist; M-H: Mantel-Hanzel; QUOROM: quality of reporting of meta-analyses; RR: relative risk; SALM: salmeterol; SFC: salmeterol/fluticasone combination; TORCH: TOwards a Revolution in COPD Health; UPLIFT: Understanding Potential Long-Term Impacts on Function with Tiotropium

## Competing interests

AK: none to declare

LDL has grant-in-aid from AstraZeneca

DDS has received honoraria for speaking engagements from GlaxoSmithKline (GSK), AstraZeneca (AZ) and Pfizer, and research funding from GSK, AZ, Wyeth Pharmaceuticals, Boehringer Ingelheim, and Pfizer over the last 3 years.

## Authors' contributions

DDS conceived the idea, designed the study, acquired the primary data, performed the statistical analysis and wrote the paper.

AK acquired the primary data and wrote the paper

LDL participated in the study design, assisted on the statistical analysis, and edited the paper.

All authors have read and approved the final manuscript.

## Supplementary Material

Additional file 1 **Table S1 Detailed Electronic Search Terms Used To Identify Relevant Clinical Trials**. We used Embase^®^, Medline^® ^and Cochrane Clinical Trial Registry^® ^Databases to identify relevant clinical trials for the present study. We have included detailed search terms and the number of hits that were obtained using these search terms on their own and in combination. Click here for file
