# Supplementary material for: The effects of long-acting bronchodilators on total mortality in patients with stable chronic obstructive pulmonary disease
Source: Respir Res. 2010 May 11;11(1):56. doi: 10.1186/1465-9921-11-56 (PMC2876086; doi:10.1186/1465-9921-11-56)
Supplement: Additional file 1 — Table S1 Detailed Electronic Search Terms Used To Identify Relevant Clinical Trials. We used Embase®, Medline® and Cochrane Clinical Trial Registry® Databases to identify relevant clinical trials for the present study. We have included detailed search terms and the number of hits that were obtained using these search terms on their own and in combination. [file 1465-9921-11-56-S1.DOC]

**Table S1 - *Search Strategy and Terms***

EMBASE:

| **A. Search Terms for COPD** | **Hits** |
| --- | --- |
| 1. exp Pulmonary Disease, Chronic Obstructive | 31088 |
| 1. exp Mediastinal Emphysema/ or exp Emphysema/ or exp Subcutaneous Emphysema/ or exp Pulmonary Emphysema/ | 14071 |
| 1. exp Bronchitis | 21245 |
| 1. (chronic obstructive lung disease* or COPD or obstructive lung disease* or obstructive pulmonary disease* or chronic obstructive disease*).mp. [mp=title, abstract, subject headings, heading word, drug trade name, original title, device manufacturer, drug manufacturer name] | 34094 |
| 1. or/1-4 | 63597 |
| **B. Search Terms for drugs** |  |
| 1. (formoterol or oxeze or foradil or eformoterol).mp. [mp=title, abstract, subject headings, heading word, drug trade name, original title, device manufacturer, drug manufacturer name] | 3249 |
| 1. (tiotropium or spiriva).mp. [mp=title, abstract, subject headings, heading word, drug trade name, original title,device manufacturer, drug manufacturer name] | 1230 |
| 1. (salmeterol or serevent).mp. [mp=title, abstract, subject headings, heading word, drug trade name, original title, device manufacturer, drug manufacturer name] | 5373 |
| 1. symbicort.mp. or budesonide/formoterol [mp=title, abstract, subject headings, heading word, drug trade name, original title, device manufacturer, drug manufacturer name | 348 |
| 1. advair.mp. or fluticasone/salmeterol [mp=title, abstract, subject headings, heading word, drug trade name, original title, device manufacturer, drug manufacturer name] | 324 |
| 1. (symbicort or advair).mp. [mp=title, abstract, subject headings, heading word, drug trade name, original title, device manufacturer, drug manufacturer name] | 605 |
| 1. long acting anticholinergic*.mp. [mp=title, abstract, subject headings, heading word, drug trade name, original title, device manufacturer, drug manufacturer name] | 90 |
| 1. long acting bronchodilator*.mp. [mp=title, abstract, subject headings, heading word, drug trade name, original title, device manufacturer, drug manufacturer name] | 241 |
| 1. long acting beta 2 agonist*.mp. [mp=title, abstract, subject headings, heading word drug trade name, original title, device manufacturer, drug manufacturer name | 111 |
| 1. LABA or LABAS).mp. [mp=title, abstract, subject headings, heading word, drug trade name, original title, device manufacturer, drug manufacturer name] | 275 |
| 1. or/6-15 | 7313 |
| 1. 5 and 16 | 2212 |
| 1. random*.mp. [mp=title, abstract, subject headings, heading word, drug trade name, original title, device manufacturer, drug manufacturer name] | 434717 |
| 1. (meta-analy* or meta analy* or metaanaly*).mp. [mp=title, abstract, subject headings, heading word, drug trade name, original title, device manufacturer, drug manufacturer name] | 45338 |
| 1. 18 or 19 | 464770 |
| 1. 17 and 20 | 511 |
| 1. limit 26 to human | 507 |

COCHRANE:

| **Search Terms** | **Hits** |
| --- | --- |
| 1. (chronic obstructive lung disease* or COPD or obstructive lung disease* or obstructive pulmonary disease* or chronic obstructive disease*).mp. [mp=title, abstract, full text, keywords, caption text] | 195 |
| 1. exp formoterol or oxeze or foradil or eformoterol).mp. [mp=title, abstract, full text, keywords, caption text] | 36 |
| 1. (tiotropium or spiriva).mp. [mp=title, abstract, full text, keywords, caption text] | 9 |
| 1. (salmeterol or serevent).mp. [mp=title, abstract, full text, keywords, caption text] | 40 |
| 1. symbicort.mp. or budesonide/formoterol [mp=title, abstract, full text, keywords, caption text] | 14 |
| 1. advair.mp. or fluticasone/salmeterol [mp=title, abstract, full text, keywords, caption text] | 13 |
| 1. (symbicort or advair).mp. [mp=title, abstract, full text, keywords, caption text] | 14 |
| 1. long acting anticholinergic*.mp. [mp=title, abstract, full text, keywords, caption text] | 4 |
| 1. long acting bronchodilator*.mp. [mp=title, abstract, full text, keywords, caption text] | 14 |
| 1. long acting beta 2 agonist*.mp. [mp=title, abstract, full text, keywords, caption text] | 15 |
| 1. (LABA or LABAS).mp. [mp=title, abstract, full text, keywords, caption text | 26 |
| 1. or/2-11 | 61 |
| 1. random*.mp. [mp=title, abstract, full text, keywords, caption text] | 5556 |
| 1. (meta-analy* or meta analy* or metaanaly*).mp. [mp=title, abstract, full text, keywords caption text] | 4177 |
| 1. 14 or 13 | 5567 |
| 1. (emphysema or pulmonary emphysema or mediastinal emphysema or | 75 |
| 1. (bronchitis or chronic bronchitis).mp. [mp=title, abstract, full text, keywords, caption text] | 112 |
| 1. chronic pulmonary disease*.mp. [mp=title, abstract, full text, keywords, caption text] | 15 |
| 1. 1 or 16 or 17 or 18 | 326 |
| 1. 19 and 12 | 32 |
| 1. 20 and 15 | 32 |

MEDLINE (1950 to March Week 3 2009)

| 1. exp Pulmonary Disease, Chronic Obstructive | 11225 |
| --- | --- |
| 1. exp Mediastinal Emphysema/ or exp Emphysema/ or exp Subcutaneous Emphysema/ or exp Pulmonary Emphysema/ | 18675 |
| 1. exp Bronchitis | 23685 |
| 1. (chronic obstructive lung disease* or COPD or obstructive lung disease* or obstructive pulmonary disease* or chronic obstructive disease*).mp. [mp=title, original title, abstract, name of substance word, subject heading word] | 23551 |
| 1. or/1-4 | 63065 |
| 1. (formoterol or oxeze or foradil or eformoterol).mp. [mp=title, original title, abstract, name of substance word, subject heading] | 1041 |
| 1. (tiotropium or spiriva).mp. [mp=title, abstract, name of substance, subject headings] | 392 |
| 1. (salmeterol or serevent).mp. [mp=title, original title, abstract, name of substance, word, subject heading word] | 1678 |
| 1. symbicort.mp. or budesonide/formoterol [mp=title, original title, abstract, name of substance, word, subject heading word] | 392 |
| 1. advair.mp. or fluticasone/salmeterol [mp=title, original title, abstract, name of substance, word, subject heading word] | 29 |
| 1. (symbicort or advair).mp. [mp=title, original title, abstract, name of substance, word, subject heading word] | 106 |
| 1. long acting anticholinergic*.mp. [mp=title, original title, abstract, name of substance, word, subject heading word] | 79 |
| 1. long acting bronchodilator*.mp. [mp=title, original title, abstract, name of substance, word, subject heading word] | 206 |
| 1. long acting beta 2 agonist*.mp. [mp=title, original title, abstract, name of substance, word, subject heading word] | 417 |
| 1. (LABA or LABAS).mp. [mp=title, original title, abstract, name of substance, word, subject heading word] | 251 |
| 1. or/6-15 | 3019 |
| 1. 5 and 16 | 829 |
| 1. random*.mp. [mp=title, original title, abstract, name of substance, word, subject heading word] | 569790 |
| 1. (meta-analy* or meta analy* or metaanaly*).mp. [mp= mp=title, original title, abstract, name of substance, word, subject heading word] | 36130 |
| 1. 18 or 19 | 589279 |
| 1. 17 and 20 | 380 |
| 1. limit 21 to (*all adult 19 plus years)* and humans) | 282 |
| 1. limit 22 to English | 260 |
